# Sarcopenia as a Potential Risk Factor for Denosumab-Related Osteonecrosis of the Jaw in Asian Prostate Cancer Patients with Bone Metastases

**DOI:** 10.3390/diagnostics15202635

**Published:** 2025-10-19

**Authors:** Shinobu Mizushima, Daisuke Watanabe, Kazuki Yanagida, Norikazu Kawae, Kashia Goto, Tatsuya Takagi, Hajime Kajihara, Akio Mizushima

**Affiliations:** 1 Department of Palliative Medicine, Graduate School of Medicine, Juntendo University, Tokyo 113-8421, Japan; sara@juntendo.ac.jp (S.M.); n.kawae.sh@juntendo.ac.jp (N.K.); k.goto.ew@juntendo.ac.jp (K.G.); ttatsuya@juntendo.ac.jp (T.T.); akiom@juntendo.ac.jp (A.M.); 2 Department of Urology, Koto Hospital, Tokyo 136-0072, Japan; k.yanagida.sr@juntendo.ac.jp; 3 Department of Molecular and Cellular Therapeutics, Graduate School of Medicine, Juntendo University, Tokyo 113-8421, Japan; 4 Department of Orthopedic Surgery, Koto Hospital, Tokyo 136-0072, Japan; h.kajihara.jk@juntendo.ac.jp

**Keywords:** prostate cancer, bone metastases, denosumab-related osteonecrosis of the jaw, sarcopenia, body composition, inflammatory biomarkers

## Abstract

**Background/Objectives**: Denosumab-related osteonecrosis of the jaw (DRONJ) is a serious complication in patients receiving long-term antiresorptive therapy for bone metastases from prostate cancer. While established risk factors include invasive dental procedures and poor oral health, the role of body composition, with a particular focus on sarcopenia and inflammatory biomarkers, remains unclear. This study aims to evaluate the association between skeletal muscle mass, fat distribution, and systemic inflammatory biomarkers with DRONJ risk in Asian prostate cancer patients with bone metastases. **Methods**: This retrospective study reviewed 64 patients who received denosumab between 2014 and 2023. Baseline CT scans were used to measure total psoas muscle index (TPI), visceral fat area (VFA), subcutaneous fat area (SFA), and body mass index (BMI). Neutrophil-to-lymphocyte ratio (NLR) and platelet-to-lymphocyte ratio (PLR) were calculated from blood counts. Group comparisons used the Wilcoxon rank-sum or chi-squared test, and correlations were assessed using Spearman’s coefficient. **Results**: Twelve patients (18.8%) developed DRONJ, with a mean onset time of 20.3 months. The prevalence of sarcopenia was significantly higher in the DRONJ group compared to the non-DRONJ group (*p* = 0.0331). VFA, SFA, BMI, diabetes, and visceral obesity were not significant predictors. NLR, but not PLR, showed a significant negative correlation with TPI (ρ = −0.2487, *p* = 0.0475), but no direct association with DRONJ, suggesting an indirect effect via sarcopenia. **Conclusions**: Sarcopenia may be an independent risk factor for DRONJ. Inflammatory biomarkers, particularly NLR, may contribute indirectly through reduced muscle mass. Body composition assessment may improve DRONJ risk stratification.

## 1. Introduction

Denosumab is a fully human monoclonal antibody that targets the receptor activator of nuclear factor-κB ligand (RANKL), a key regulator of osteoclast formation, activation, and survival [1]. It is widely used to inhibit the progression of bone metastases in prostate cancer and to prevent skeletal-related events (SREs). Osteoclasts originate from hematopoietic stem cells, and their differentiation and function require macrophage colony-stimulating factor (M-CSF) and RANKL, which are secreted by bone marrow stromal cells and osteoblasts [2]. RANKL is essential not only for the fusion of immature osteoclasts, but also for the activation and maintenance of mature osteoclasts. By binding to RANKL with high specificity and affinity, denosumab effectively blocks the RANK–RANKL interaction, thereby suppressing osteoclast-mediated bone resorption. This mechanism not only reduces skeletal complications, but also helps maintain bone integrity in patients with advanced malignancies.

In diseases such as metastatic bone tumors and osteoporosis, where excessive bone resorption plays a central role in pathophysiology, targeting osteoclast differentiation and function is a major therapeutic strategy. Prostate cancer frequently metastasizes to the bone, with approximately 90% of advanced cases exhibiting bone metastases [3]. Therefore, suppressing osteoclast activity is essential for maintaining bone structural integrity, preventing complications such as pain and fractures, and ultimately sustaining the quality of life (QOL) and prognosis of patients with bone-metastatic prostate cancer. Denosumab has demonstrated superior renal safety and greater efficacy in preventing skeletal events compared to previously used bisphosphonates, contributing to its rapidly increasing adoption in clinical practice [4]. However, medication-related osteonecrosis of the jaw (MRONJ) has emerged as a serious adverse effect associated with systemic antiresorptive therapy. MRONJ is characterized by persistent exposure of the jawbone and refractory infection, and it often proves difficult to treat, significantly impacting patients’ QOL [5].

Although the precise mechanisms underlying MRONJ are not fully understood, they are believed to involve a combination of factors, including impaired bone remodeling, inhibition of angiogenesis, repeated microtrauma, and compromised protective functions of both bone and soft tissues [6]. Osteonecrosis of the jaw (ONJ) was first reported in patients receiving bisphosphonate (BP) therapy [7], and the incidence of bisphosphonate-related ONJ (BRONJ) subsequently increased. More recently, denosumab-related ONJ (DRONJ) has been reported and is garnering increasing attention [8]. Historically, local oral factors such as tooth extraction, ill-fitting dentures, and poor oral hygiene have been considered the primary risk factors for DRONJ [9]. These factors can directly induce trauma or infection, leading to osteonecrosis. However, recent research has increasingly emphasized the importance of systemic host-related factors, such as bone metabolism, immune function, and vascular environment, in DRONJ pathogenesis [10].

Systemic conditions, including advanced age, diabetes, smoking, and concomitant use of corticosteroids or anticancer drugs, have been associated with increased DRONJ risk, supporting the notion of a multifactorial etiology [5]. Among these, body composition has gained attention as a potential risk factor. Body composition reflects the overall physiological condition of the individual and is influenced by aging, cachexia, hormonal status, and inflammation. It is closely associated with bone metabolism, wound healing, and immune function [11,12]. Specifically, sarcopenia—characterized by the loss of skeletal muscle mass due to aging or disease—impairs bone formation and immune response via reduced secretion of muscle-derived cytokines (myokines), negatively affecting the bone repair process [13]. In parallel, accumulation of visceral fat promotes chronic systemic inflammation and may delay healing or contribute to necrosis of the jawbone by inhibiting angiogenesis and cellular regeneration [14]. These findings suggest that both reduced muscle mass and increased visceral fat are potential systemic risk factors for DRONJ.

Despite this, few clinical studies have systematically investigated the association between body composition and DRONJ risk in patients with prostate cancer. Notably, prostate cancer patients with bone metastases who receive denosumab for prolonged periods are particularly vulnerable to developing sarcopenia and abnormal fat distribution due to aging, malnutrition, and hormonal therapy, and they are, at the same time, at high risk for DRONJ. In this study, we examine body composition as a clinical risk factor for DRONJ, focusing on quantitative metrics such as sarcopenia, visceral fat, and subcutaneous fat. Our goal is to improve our understanding of previously underappreciated systemic risk factors and contribute to more refined risk stratification in clinical practice.

## 2. Materials and Methods

### 2.1. Ethical Approval

Patient consent was waived due to the retrospective design of the study, which was conducted under an opt-out policy approved by the institutional ethics committee.

### 2.2. Study Population and Data Collection

From September 2014 to August 2023, we conducted a retrospective review of 64 patients with prostate cancer and bone metastases who received denosumab at monthly doses of 120 mg. All patients received denosumab for the management of bone metastases from prostate cancer. No patient in this cohort had prior or concomitant exposure to bisphosphonates or other bone-modifying agents. Baseline data were obtained from electronic medical records and included age, height, weight, body mass index (BMI), Gleason score, and the presence of diabetes mellitus, hypertension, and current smoking status.

Laboratory data collected 1–3 weeks before denosumab initiation included neutrophil, lymphocyte, and platelet counts, serum albumin, serum calcium, prostate-specific antigen (PSA), and C-reactive protein (CRP). The neutrophil-to-lymphocyte ratio (NLR) and platelet-to-lymphocyte ratio (PLR) were calculated as indicators of systemic inflammation and immunonutritional status.

### 2.3. CT-Based Body Composition Measurements

At the time of prostate cancer diagnosis, CT scans performed for metastatic work-up were used to assess the psoas muscles at the level of the third lumbar vertebra (L3) by manual tracing. The total psoas muscle index (TPI) was calculated as the sum of the cross-sectional areas of both psoas muscles divided by the square of the patient’s height (cm^2^/m^2^) and was used as an indicator of skeletal muscle mass. Visceral fat area (VFA) and subcutaneous fat area (SFA) were measured at the umbilical level using CT images processed with SYNAPSE VINCENT imaging software (Fujifilm Medical, Tokyo, Japan). Sarcopenia was defined according to previously validated TPI cut-off values for Asian populations: ≤3.46 cm^2^/m^2^ for females and ≤4.78 cm^2^/m^2^ for males [15]. Visceral obesity was defined as VFA ≥ 100 cm^2^, based on the criteria of the Japan Society for the Study of Obesity [16].

### 2.4. Statistical Analysis

Values are presented as median (interquartile range) for non-normally distributed continuous variables, mean ± standard deviation (SD) for normally distributed continuous variables, or number (%) for categorical variables. Differences between patients who developed DRONJ and those who did not were assessed using the Wilcoxon rank-sum test for continuous variables and the chi-squared test for categorical variables, as appropriate. Spearman’s rank correlation coefficient was used to evaluate the associations between TPI and inflammatory markers, including NLR and PLR. *p*-values of <0.05 were considered to indicate statistical significance. All statistical analyses were performed using the JMP Pro 18 software program (SAS Institute Inc., Cary, NC, USA).

## 3. Results

### 3.1. Patients Characteristics

A total of 64 patients with prostate cancer and bone metastases who received denosumab were included in this analysis (Table 1). The median age was 75.1 years (range, 54–89) and the median BMI was 21.7 kg/m^2^ (IQR, 19.8–23.4). Diabetes mellitus and hypertension were present in 20.3% and 40.6% of patients, respectively, and 30.0% were current smokers. The majority of patients (75.0%) had a Gleason score of ≥8. Median PSA was 247.9 ng/mL (IQR, 64.9–1127.5). Mean TPI was 6.2 ± 1.6 cm^2^/m^2^, and sarcopenia was observed in 14.1% of patients. Mean VFA and SFA were 118.0 ± 64.8 cm^2^ and 100.0 ± 59.5 cm^2^, respectively. Out of 64 patients, 12 (18.8%) developed DRONJ, with a mean onset time of 20.3 months after initiation of denosumab therapy.

### 3.2. Comparison Between Patients with and Without DRONJ

When comparing clinical characteristics between patients who developed DRONJ and those who did not (Table 2), the prevalence of sarcopenia was significantly higher in the DRONJ group (33.3% vs. 9.6%, *p* = 0.0331). No significant differences were observed between the two groups in relation to age, BMI, diabetes mellitus, hypertension, current smoking, Gleason score, PSA, VFA, SFA, NLR, or PLR.

### 3.3. Correlation Analyses

**Figure 1:**
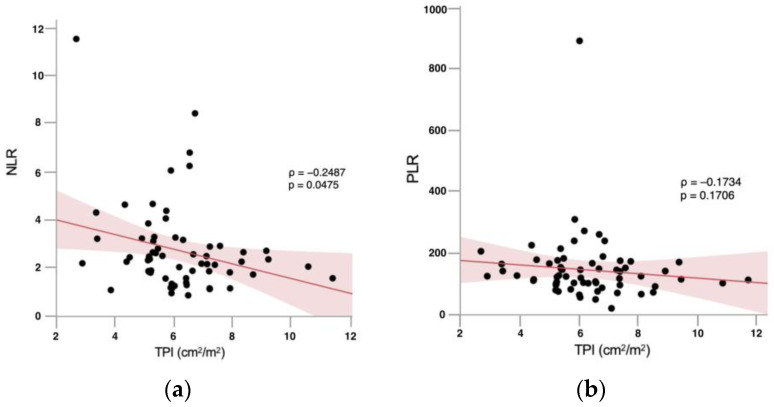
Spearman’s rank correlation coefficients illustrating the association between total psoas muscle index (TPI) and inflammatory biomarkers. (**a**) An inverse correlation was observed between neutrophil-to-lymphocyte ratio (NLR) values and TPI (Spearman’s Rho = −0.2487, *p* = 0.0475). (**b**) A tendency toward a positive association between platelet-to-lymphocyte ratio (PLR) values and TPI was observed; however, the correlation did not reach statistical significance (Spearman’s Rho = −0.1734, *p* = 0.1706).

Spearman’s rank correlation analysis revealed a significant inverse correlation between NLR and TPI (ρ = −0.2487, *p* = 0.0475), indicating that lower NLR values were associated with lower skeletal muscle mass and a higher prevalence of sarcopenia (Figure 1). In contrast, PLR showed a weak non-significant negative correlation with TPI (ρ = −0.1734, *p* = 0.1706) (Figure 1).

## 4. Discussion

In this Asian cohort of prostate cancer patients with bone metastases receiving denosumab, sarcopenia emerged as a potential risk factor for DRONJ. At the start of denosumab therapy, the prevalence of sarcopenia was 14.1%. For context, a meta-analysis of community-dwelling Chinese older adults reported a pooled prevalence of 12.9% in men [17]. Thus, our estimate appears comparable to, at most, slightly higher than population-based figures rather than demonstrably higher. Small differences are likely attributable to methodological and population differences, including the use of CT-derived total psoas muscle index (TPI) in our study versus DXA-derived appendicular lean mass commonly used in population studies, as well as the mixed East Asian composition (Japanese, Chinese, Korean) and the advanced cancer context of our cohort. Importantly, body composition was assessed on CT obtained before the first denosumab administration and immediately before or at the initiation of androgen deprivation therapy (ADT); therefore, cumulative ADT exposure did not influence the baseline prevalence estimate in this study. However, whether ADT per se modified DRONJ risk in this cohort remains uncertain because ADT and denosumab were co-administered in all cases.

Denosumab is a human monoclonal antibody against RANKL that inhibits osteoclast differentiation and function and is widely used for the prevention of SREs in osteoporosis and bone metastases [18]. While its potent antiresorptive effect contributes to its therapeutic efficacy, denosumab is also known as a major causative agent of MRONJ, making prevention and early detection of MRONJ important clinical challenges. Previous clinical trials have reported the incidence of DRONJ in cancer patients to be between 0.7% and 2.0% [19,20]. However, real-world data from Bracchi et al. demonstrated higher cumulative incidence rates, with 5.7% at 24 months and 9.8% at 48 months [21]. In prostate cancer patients in particular, treatment for bone metastases often extends over a long period, leading to an increased cumulative dose of denosumab, which may result in a relatively higher risk of developing DRONJ. Furthermore, a study by Yoshimura et al. on patients with metastatic prostate cancer suggested that DRONJ presents a clinical picture similar to that of BRONJ and is more frequently observed in elderly male patients and those with a history of tooth extraction [22]. A nationwide cohort study conducted in South Korea by Kim et al. reported that individuals aged 80 years or older had a threefold higher risk of developing ARONJ compared to those aged 50–59, indicating that advanced age is an independent risk factor for ARONJ [23]. These findings suggest that DRONJ may develop as a result of multiple interrelated risk factors, including dosage, duration of treatment, oral invasive procedures, and patient-specific background characteristics. They underscore the need for careful risk management, particularly in patients with prostate cancer.

Various pathological conditions have been reported to be associated with MRONJ. An association between obesity and an increased risk of ONJ has been reported. Although the mechanisms by which obesity contributes to ONJ remain largely unclear, potential explanations include steroid-induced weight gain and the possibility that obese patients may have longer survival and therefore receive more prolonged chemotherapy and steroid treatment [24]. Moreover, obese patients have been shown to exhibit elevated circulating levels of various cytokines and acute-phase proteins associated with inflammation [25]. Adipocytes have also been reported to secrete several cytokines and acute-phase proteins, thereby directly or indirectly increasing the production and circulation of factors involved in the inflammatory process [26]. The study by Wilkinson et al. suggests that inflammatory lesions in the jaw constitute a significant component of the pathogenesis of BRONJ and DRONJ and highlights the critical importance of early detection and management of inflammation in the prevention of ONJ [27]. This report states that the causes of ONJ involve infectious conditions, impaired blood flow to the bone, and excessive suppression of bone metabolism—all of which are closely associated with chronic inflammation. It also suggests that avoiding local inflammation caused by dental interventions, such as tooth extraction, may contribute to risk reduction.

In recent years, diabetes mellitus (DM) has gained attention as one of the systemic risk factors for MRONJ. Epidemiologically, several case–control and observational studies have reported a higher prevalence of diabetes mellitus among patients with MRONJ compared to those without the condition. For example, Molcho et al. reported that 58% of patients with MRONJ had diabetes mellitus or impaired fasting glucose, whereas only 12% of bisphosphonate users without MRONJ exhibited these conditions [28]. Furthermore, the report by Watters et al. indicated that the presence of diabetes was associated with a poorer clinical course of MRONJ, suggesting its potential involvement in disease progression [29]. Although some variability has been described among earlier studies, more recent reports have supported the same trend. For instance, a nationwide analysis from Austria demonstrated that DM and hyperglycemia significantly increased the risk of MRONJ [30]. Collectively, both historic and contemporary evidence indicates that diabetes mellitus should be regarded as a potential systemic risk factor for MRONJ [31,32,33].

From a pathophysiological perspective, diabetes is thought to contribute to the onset and progression of MRONJ through multiple pathways. Hyperglycemia-induced microvascular damage and endothelial dysfunction impair local blood flow and angiogenesis in bone tissue, thereby hindering the healing process. In addition, the accumulation of advanced glycation end-products (AGEs) inhibits the adhesion and differentiation of osteoblasts and osteocytes while promoting apoptosis [34,35]. Furthermore, diabetes is associated with altered immune function, including impaired neutrophil chemotaxis and phagocytosis, reduced macrophage activity, and excessive production of proinflammatory cytokines, all of which increase the risk of infection and chronic inflammation [36]. The interplay of these factors creates an environment unfavorable for bone healing, thereby increasing the susceptibility to MRONJ during antiresorptive therapy. Moreover, genetic polymorphisms in drug-metabolizing enzymes associated with diabetes—particularly mutations in CYP2C8—have been suggested to influence the metabolism of thiazolidinediones and bone metabolic pathways, potentially enhancing the individual susceptibility to medication-related osteonecrosis of the jaw [37].

Although visceral obesity and diabetes mellitus have be suggested as systemic risk factors for MRONJ in previous studies, neither was identified as a significant predictor of DRONJ in our cohort. This discrepancy may be partly attributable to the limited sample size and statistical power, differences in patient background, and the relatively good systemic and oral management provided at our institution. In our cohort, NLR—but not PLR—showed a significant negative correlation with TPI, suggesting that elevated inflammatory status, as reflected by NLR, may be linked to reduced skeletal muscle mass. However, neither NLR nor PLR demonstrated a direct statistically significant association with DRONJ occurrence. These findings imply that inflammation-related markers, particularly NLR, may act as indirect risk factors for DRONJ, potentially through their association with sarcopenia, rather than serving as independent predictors. This underscores the interplay between systemic inflammation, muscle mass, and susceptibility to skeletal complications in patients receiving long-term antiresorptive therapy. Importantly, to our knowledge, this is among the first studies to evaluate DRONJ risk in Asian patients with metastatic prostate cancer based on clinical body composition parameters, such as skeletal muscle mass and fat distribution. Our findings highlight the potential importance of sarcopenia and inflammation-related markers, such as NLR and PLR, in DRONJ risk stratification. These observations warrant validation in larger, prospective, multicenter studies.

This study has several limitations. First, it was a retrospective analysis conducted at a single institution with a relatively small sample size, which may limit the generalizability of the findings. Second, the diagnosis of DRONJ was based on clinical and radiological findings made at affiliated hospitals, including university hospitals with oral and maxillofacial surgery departments, as our institution does not have such a department. Histopathological confirmation was not obtained in all cases, which may have introduced misclassification bias. Third, the presence of sarcopenia at baseline likely reflects multifactorial contributors, including advanced age, cancer-related catabolism, prior exposure to androgen deprivation therapy (ADT), nutritional status, and lifestyle factors. Given the retrospective design, we were unable to quantify the relative contribution of each factor; this remains an important target for prospective standardization. Fourth, bone mineral density (BMD) was not systematically assessed in this cohort, as dual-energy X-ray absorptiometry (DXA) was not routinely performed. The absence of BMD data limited our ability to evaluate the interplay between osteoporosis, sarcopenia, and DRONJ risk. Future studies should integrate BMD measurements alongside body composition analysis. Fifth, variations in dental management prior to and during denosumab therapy were not fully controlled for, potentially affecting DRONJ incidence. Sixth, although TPI was generally assessed using CT scans obtained before the first denosumab administration, minor variations in timing relative to prostate cancer diagnosis and initiation of systemic therapy may have occurred. This lack of full homogeneity could have introduced variability in baseline muscle mass estimation. Future studies should include larger, multicenter prospective cohorts to validate the association between sarcopenia and DRONJ risk in prostate cancer patients with bone metastases. Incorporating standardized oral health assessments and longitudinal body composition measurements may help to clarify causal relationships. Furthermore, interventional trials aimed at maintaining or improving skeletal muscle mass could provide insight into preventive strategies for DRONJ in this patient population.

## 5. Conclusions

In this retrospective study of Asian prostate cancer patients with bone metastases receiving denosumab, pre-existing sarcopenia diagnosed before the initiation of denosumab therapy emerged as a potential risk factor for DRONJ, whereas visceral obesity and diabetes mellitus were not significant predictors. Inflammation-related markers such as NLR and PLR correlated with reduced skeletal muscle mass and may represent indirect risk factors for DRONJ through their association with sarcopenia. These findings highlight the need for prospective validation and suggest that maintaining skeletal muscle mass may represent a promising preventive strategy in this high-risk population.

## Figures and Tables

**Table 1:** Baseline characteristics.

Variables		Overall Patients
Total	No. (%)	64 (100)
Age	Mean (range)	75.1 (54–89)
Ethnicity		
Japanese	No. (%)	60 (93.7)
Chinese	No. (%)	3 (4.7)
Korean	No. (%)	1 (1.6)
Height (cm)	Median (IQR)	162 (157−168)
Body weight (kg)	Median (IQR)	56 (49.3−65.8)
BMI (kg/m^2^)	Median (IQR)	21.7 (19.8−23.4)
Diabetes mellitus	No. (%)	13 (20.3)
Hypertension	No. (%)	26 (40.6)
Current smoking	No. (%)	19 (30.0)
Gleason score		
6	No. (%)	1 (1.6)
7	No. (%)	15 (23.4)
≥8	No. (%)	48 (75.0)
PSA (ng/mL)	Median (IQR)	247.9 (64.9−1127.5)
Neutrophils (10^3^/mm^3^)	Mean ± SD	4.34 ± 1.93
Lymphocytes (10^3^/mm^3^)	Mean ± SD	1.9 ± 0.72
Platelets (10^3^/mm^3^)	Mean ± SD	219.0 ± 65.2
NLR	Mean ± SD	2.7 ± 1.8
PLR	Mean ± SD	139.8 ± 110.0
TPI (cm^2^/m^2^)	Mean ± SD	6.2 ± 1.6
Sarcopenia	No. (%)	9 (14.1)
SFA (cm^2^)	Mean ± SD	100.0 ± 59.5
VFA (cm^2^)	Mean ± SD	118.0 ± 64.8
DRONJ	No. (%)	12 (18.8)
Time to DRONJ onset (months)	Mean ± SD	20.3 ± 8.5

Values are presented as number (%) for categorical variables, median (interquartile range) for non-normally distributed continuous variables, and mean ± standard deviation (SD) for normally distributed continuous variables.

**Table 2:** Comparison of clinical characteristics between patients with and without DRONJ.

	Group A (*n* = 12)	Group B (*n* = 52)	*p*
Median age, years (IQR)	76 (69.8–79.8)	76.5 (71–80)	0.6543
Median BMI, kg/m^2^ (IQR)	20.5 (19.5–21.7)	22.0 (19.8–24.2)	0.1196
Diabetes mellitus, *n* (%)	3 (25.0)	10 (19.2)	0.6543
Hypertension, *n* (%)	4 (33.3)	22 (42.3)	0.5683
Current smoking, *n* (%)	3 (25.0)	16 (30.8)	0.6934
Sarcopenia, *n* (%)	4/12 (33.3)	5/52 (9.6)	0.0331
Median VFA, cm^2^ (IQR)	95.8 (48.9–120.8)	117.6 (73.1–170.4)	0.2527
Visceral obesity, *n* (%)	5 (41.7)	29 (55.8)	0.3775
Median SFA, cm^2^ (IQR)	75.3 (50.2–133.8)	96.1 (52.3–153.1)	0.2527
Median PSA, ng/mL (IQR)	199.9 (43.9–1643)	263.7 (73.5–1030.7)	0.7634
Median Gleason score (IQR)	8 (7.3–9)	8 (7.3–9)	0.5122
Median NLR, (IQR)	2.7 (1.4–3.8)	2.2 (1.7–2.8)	0.3143
Median PLR, (IQR)	136.6 (110.1–165.2)	113.4 (90.2–162.4)	0.2901

Group A: Patients who developed DRONJ. Group B: Patients who did not develop DRONJ. Values are presented as median (interquartile range) or number (%). Differences between groups were assessed using the Wilcoxon rank-sum test or the chi-squared test, as appropriate.

## Data Availability

The original contributions presented in this study are included in the article. Further inquiries can be directed to the corresponding author.
